# Robotic Single-Site Cholecystectomy: A Single-Center Retrospective Study

**DOI:** 10.7759/cureus.50271

**Published:** 2023-12-10

**Authors:** Naved Salim, Camryn Daidone, Leslie Smith, Ahsan Raza

**Affiliations:** 1 Research, Edward Via College of Osteopathic Medicine, Shreveport, USA; 2 General Surgery, Rapides Regional Medical Center, Alexandria, USA

**Keywords:** chronic cholecystitis, gall bladder, laparoscopic, robotic, single-site cholecystectomy

## Abstract

Objective

Our goal is to describe and report the outcomes of patients undergoing robotic single-site cholecystectomy at a single institution.

Background

Cholecystectomy is a common procedure performed to remove the gallbladder. Robotic single-site cholecystectomy (RSSC) is rapidly emerging as a safe and feasible alternative to conventional 4-port laparoscopic techniques. Patients who undergo RSSC procedures may also have a decreased need for postoperative analgesics and a lower postoperative pain score.

Methods

From September 2020 to August 2023, there were 33 cases of RSSC performed by a single surgeon at a single institution. We recorded demographic data including sex, age, and BMI as well as postoperative outcome data including wound dehiscence, postoperative infection, biliary leakage, wound herniation, blood loss, and conversion to open procedure.

Results

The patient group included 24 females (72.7%) and nine males (27.3%) with a median age of 32 (Range: 9-70) and a median BMI of 24.2 kg/m^2^ (Range: 18.1-30.7). The majority of these patients were receiving cholecystectomies for benign conditions such as symptomatic cholelithiasis (n = 18, 54.5%), biliary dyskinesia (n = 13, 39.4%), acute cholecystitis (n = 1, 0.03%), and biliary colic (n = 1, 0.03%). The average estimated blood loss was 5.91 mL. Thirty-two patients (96.9%) were discharged home the same day of surgery. One patient was admitted overnight for observation due to severe biliary dyskinesia diagnosed preoperatively. The patient had no complications and was discharged the following day. One patient presented with acute abdominal wall cellulitis and omphalitis with no underlying abscess four weeks after the operation. They were treated with therapeutic antibiotics. No patients underwent conversion to an open procedure. There were no incidences of postoperative wound dehiscence or biliary leakages. One patient was admitted overnight for observation of biliary dyskinesia and another experienced abdominal wall cellulitis four weeks post-operation.

Conclusions

Although conventional multi-incision laparoscopic cholecystectomy remains the gold standard treatment for benign gallbladder disease, our study demonstrates the practicality and safety of Robotic Single-Site Cholecystectomy procedures.

## Introduction

A cholecystectomy is a very common procedure performed to remove the gallbladder due to benign or malignant conditions of the gallbladder and biliary tree. Open cholecystectomy procedures were traditionally used for the treatment of gallbladder disease. However, open procedures have a higher risk for infection, wound dehiscence, herniation, respiratory complications, higher pain scores, and longer postoperative stays. In the last two decades, there has been a significant push towards the use of laparoscopic techniques for cholecystectomy. Due to its success and favorable postoperative outcomes, laparoscopic cholecystectomy is now the gold standard for gallbladder removal, particularly for benign gallbladder disease [1]. Robotic cholecystectomy emerged as a safer and more feasible procedure when compared to laparoscopic procedures [2]. This procedure requires multiple incision sites for a camera as well as the four robot arms needed to perform this minimally invasive surgery. Robotic cholecystectomy was associated with more favorable postoperative outcomes such as shorter duration of stay and lower readmission rates [2]. Now, a new and even less invasive technique is emerging in an attempt to further improve outcomes for patients undergoing removal of the gallbladder. Robotic single-site cholecystectomy (RSSC) is rapidly emerging as a safe and feasible alternative to conventional 4-port laparoscopic techniques [3-5]. Current research suggests significant benefits of a single-incision for operative use including improved cosmetics and shorter hospital stays [6], but more research is needed to investigate the efficacy of RSSC and which patient populations are most suited for this procedure.

In a robotic single-site cholecystectomy, a single 2 to 3-cm transumbilical skin incision is made for the insertion of all 3 or 4 trocars, negating the need for multiple incisions as in traditional laparoscopic techniques. The use of single-incisions for surgery has been associated with significantly less bleeding, lower rates of postoperative complications, and more favorable cosmetics [7]. Patients who undergo RSSC procedures also have a decreased need for postoperative analgesics and a lower postoperative pain score [8]. Additionally, research suggests that the learning curve for RSSC procedures in a surgeon who is already trained to perform conventional laparoscopic cholecystectomies is minimal, making it a practical alternative [9,10].

Our goal is to report on the demographics and surgical outcomes of patients undergoing robotic single-site cholecystectomy in order to determine the feasibility of this procedure when compared to conventional cholecystectomy procedures. We hope that this study will contribute to a body of literature evaluating the efficacy of RSSC in treating gallbladder conditions and improving postoperative outcomes.

## Materials and methods

For our retrospective study, we gathered information from patients who underwent RSSC procedures by a single surgeon at a single institution in the last three years. From September 2020 to August 2023, there were 33 total cases and a majority of our patients underwent RSSC procedures for symptomatic cholelithiasis and biliary dyskinesia. Patients were not offered the RSSC procedure if they had acute cholecystitis, previous abdominal surgeries (previous scar tissue), and a BMI > 30 kg/m^2^ (the gel port mentioned below was not large enough to accommodate patients with higher abdominal fat content). We recorded demographic data including sex, age, and BMI, and also postoperative outcome data including wound dehiscence, postoperative infection, biliary leakage, wound herniation, blood loss, and conversion to open procedure.

Surgical procedure

**Figure 1:**
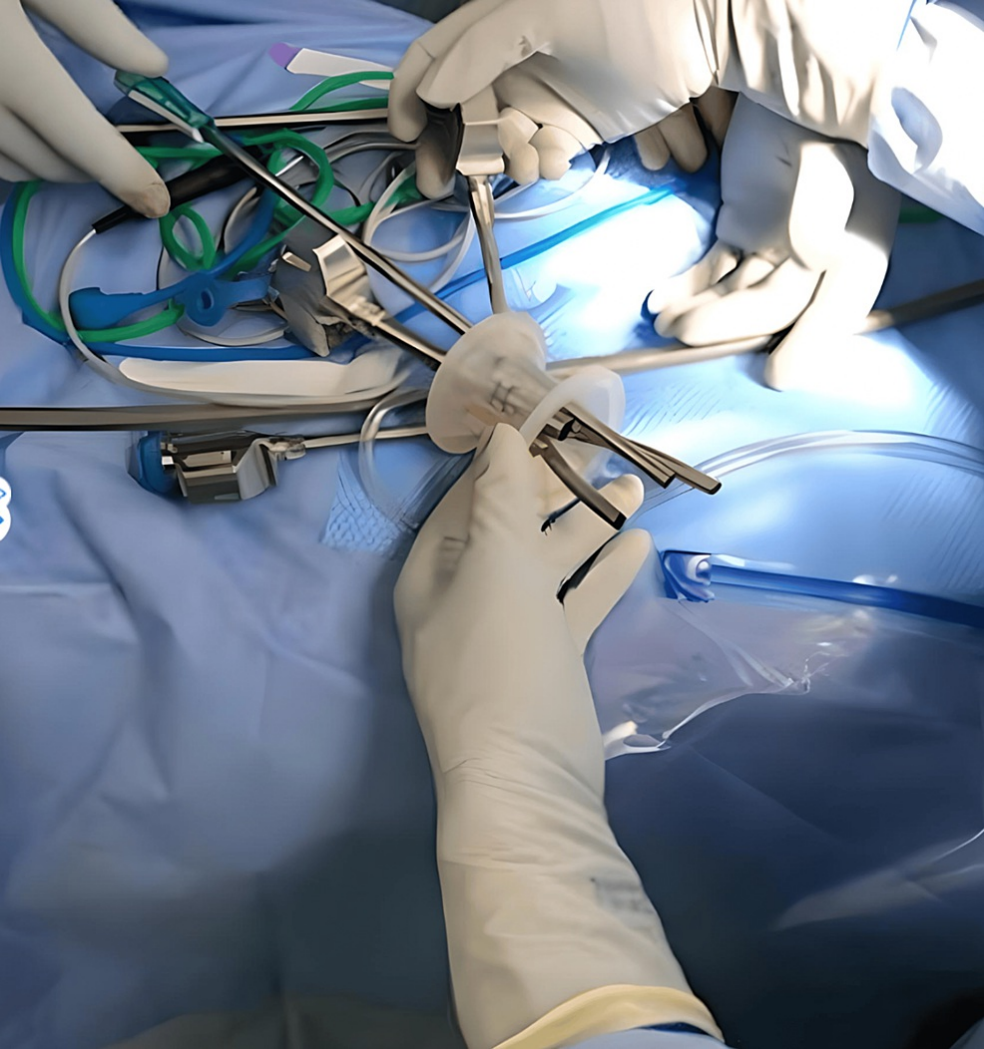
Single-site gel point (gray tube) with three other robotic trocar arms inserted

**Figure 2:**
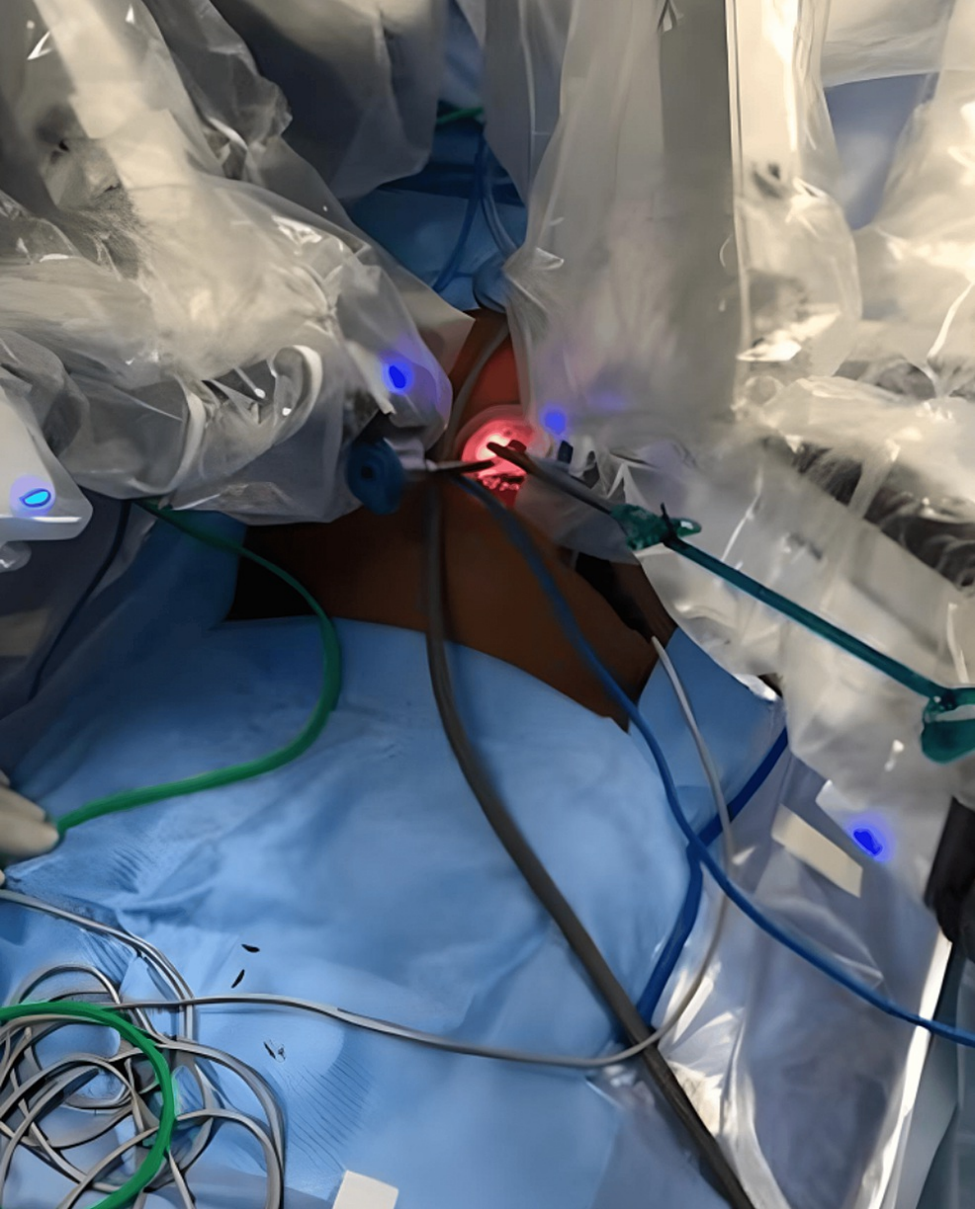
All robotic arms inserted through the single-site gel point via umbilical incision site

Each patient was placed in a supine position with both arms tucked and the abdomen prepped and draped in a sterile fashion. A pre-procedure time-out identifying the patient by their name, age, medical number, and correct procedure was performed. Perioperative antibiotics were administered and the peri-umbilical area was infiltrated with local anesthetic and a 2 to 3-cm vertical incision. After dissecting the subcutaneous tissue and fascia, an incision was made and a single-site gel point was inserted, followed by insufflation of the abdomen up to 15 mmHg (Figures 1, 2). Once this was done, the camera port was inserted and the abdomen was visualized. The robot was docked and the surgical and assistant trocars were inserted under direct visualization from the right and left side. The gallbladder was targeted and the patient was positioned tilted to the left while still in the supine position to allow for better visualization. With the help of a fenestrated bipolar and hook cautery, any adhesions, if present, were gently taken down. IndoCyanine Green (ICG) imaging was used on all patients to identify biliary anatomy. After 2 mL of ICG was pushed, the Firefly system was activated, allowing visualization of anatomical sections that still had blood flow. This was used in all patients as an extra precaution in order to not dissect anything with active blood flow and cause more blood loss. After careful dissection, the cystic duct and cystic artery were identified separately. Calot's triangle was identified and a Hepatocystic Triangle of Safety view was obtained. Clips were introduced from the right arm and three clips were each placed, two proximal and one distal, on the cystic artery and the cystic duct. This was then divided with scissors and a bile leakage test was performed to make sure there was no leak from any stump. The cautery was again used to dissect the gallbladder off the liver bed making sure that hemostasis was achieved throughout with gentle traction from the left arm. Once the gallbladder was completely separated from the liver bed, it was placed into a specimen bag for removal. The robot was then undocked and the gallbladder was retrieved along with the gel port sites, from the transumbilical incision. The incision was treated with local anesthetic and closed with a 3-0 Polydioxanone Suture (PDSTM) stitch. The skin incision was then infiltrated with local anesthetic and closed with a 4-0 Monocryl^TM^ suture and Dermabond^TM^ was applied.

## Results

Our patient population included 24 females (72.7%) and nine males (27.3%) with a median age of 32 (Range: 9-70) and a median BMI of 24.2 kg/m^2^ (Range: 18.1-30.7 kg/m^2^). The majority of these patients were receiving cholecystectomies for benign conditions such as symptomatic cholelithiasis (n = 18, 54.5%), biliary dyskinesia (n = 13, 39.4%), acute cholecystitis (n = 1, 0.03%), and biliary colic (n = 1, 0.03%) (Table 1). The average estimated blood loss was 5.91 mL and the median blood loss was 5 mL. Thirty-two patients (96.9%) were discharged home the same day of surgery. One patient was admitted overnight for observation due to severe biliary dyskinesia diagnosed preoperatively. This patient had no complications and was discharged the following day. One patient presented with acute abdominal wall cellulitis and omphalitis with no underlying abscess four weeks after the operation. They were treated with therapeutic antibiotics. No patients underwent conversion to an open procedure. There were no incidences of postoperative wound dehiscence, hernia, or biliary leakages. One patient was admitted overnight for observation of biliary dyskinesia and another experienced abdominal wall cellulitis four weeks post-operation.

**Table 1:** Preoperative diagnoses of 33 patients undergoing robotic single-site cholecystectomy

Preoperative Diagnosis	Number of Patients
Cholelithiasis	18 (54.5%)
Biliary Dyskinesia	13 (39.4%)
Acute Cholecystitis	1 (0.03%)
Biliary Colic	1 (0.03%)

## Discussion

Of our 33 patients who underwent Robotic Single-Site Cholecystectomy, no patients experienced postoperative hernia, wound dehiscence, or biliary leakages. Patients were instructed to follow up at the clinic 3-4 weeks after their operation or sooner if any complications arose. No wound herniations were noted, however, postoperative hernias are normally a delayed complication of surgery so a longer follow-up is needed to determine whether any of our patients developed this complication. There was one patient out of the 33 patient samples that presented with acute abdominal wall cellulitis and omphalitis with no underlying abscess four weeks after the operation. No other patients experienced any post-operative infections. None of these patients underwent conversion to an open procedure nor did any of the surgeries require extra trochar insertions.

Our patient demographic was mostly consistent with the stereotypical patient population for benign disease of the gallbladder [11,12]. In our study, 24 out of 33 of our patients were female and the average age of our patient population is 37.64 years old. However, while obesity is a strong risk factor for benign gallbladder disease, the BMI of patients in our study ranged from 18.1 to 30.7 kg/m^2^ with a median of 24.2 kg/m^2^. This value falls within the ranges for normal weight (Normal range: 18.5-24.5; Overweight: 25.0-29.9). The smaller average BMI of the patients in this study is due to the physical limitations of using a single-site trocar gel port. This specific gel port can only accommodate for approximately 2 inches of subcutaneous fat.

The median estimated blood loss across all of our 33 patients was 5 mL. This lower average aligns with a case series done at community hospitals in Hawai’i, which demonstrated an average estimated blood loss of 33.7 (+/- 27.4) mL for single-incision laparoscopic cholecystectomies (SILC) among 130 patients [13]. This supports the use of Robotic Single-Site Cholecystectomy as an equivalent procedure to laparoscopic cholecystectomy as a way to decrease blood loss during the procedure and accommodate patients who may be prone to excessive bleeding or who are less hemodynamically stable.

Patients undergoing open cholecystectomy can expect postoperative hospital stays ranging from two to six days [14]. However, as procedures become less invasive, postoperative stays decrease accordingly. One study reported an average length of postoperative hospital stay of 2.29 days among 53 patients undergoing laparoscopic cholecystectomy [15], with other similar studies reporting a length of stay between 1.1 and 3.5 days [16]. All 33 of our patients were discharged within 24 hours of their procedure with one patient being readmitted four weeks later. Therefore, our study aligns with others in supporting the use of RSSC procedures for the reduction of hospital stays and, consequently, decreased hospital costs for patients.

The involvement of only one surgeon is a significant strength of our study. This ensured a standardized RSSC procedure across all patients with no potential variations that may arise from procedures performed by different surgeons at different institutions. However, this also brought forth a limitation of our study involving a small sample size from a single surgeon at a single institution. Future studies may include larger sample sizes involving patients from different surgeons across multiple institutions.

## Conclusions

Our retrospective analysis of 33 patients at a single institution highlighted the post-operative outcomes of performing robotic single-site cholecystectomy procedures. Overall, it was noted that this procedure was as safe and effective for our patients as laparoscopic or multiple-incision procedures, resulting in favorable outcomes in the context of wound infection and dehiscence.

Conventional multi-incision laparoscopic cholecystectomy remains the gold standard treatment for benign gallbladder disease. However, our study demonstrates the practicality and safety of robotic single-site cholecystectomy procedures, leading to decreased infection, and shorter hospital stays.
